# Effects of physical activity on infertility in reproductive females

**DOI:** 10.1186/s12958-024-01234-6

**Published:** 2024-05-29

**Authors:** Hanzhi Zhang, Lan Hua, Dan Liu, Xin Su, Jianlin Chen, Jingfei Chen

**Affiliations:** Reproductive Medicine Center, Department of Obstetrics and Gynecology, The Second Xiangya Hospital, Central South University, Changsha, Hunan China

**Keywords:** Physical activity, Recreation activity, Work activity, Infertility, Lifestyle

## Abstract

**Objectives:**

To explore the relationship between different types of physical activity and female infertility.

**Methods:**

This study analyzed data from 2,796 female participants aged 18–44 years in the United States, obtained from the National Health and Nutrition Examination Survey (NHANES) database spanning the years 2013 to 2020. Multiple logistic regression analyses and generalized linear models were used to explore the relationship between different types of physical activity and infertility after adjusting for potential confounding factors.

**Results:**

We found a non-linear relationship between recreational activities and infertility with an inflection point of 5.83 h/week (moderate intensity), while work activities and traffic-related activities did not. On the left side of the inflection point, there was no significant association between recreational activity time and infertility (OR = 0.93, 95% CI: 0.86 to 1.02, *P* = 0.1146), but on the right side of the inflection point, there was a positive association between recreational activity time and the risk of infertility (OR = 1.04, 95% CI: 1.02 to 1.06, *P* = 0.0008).

**Conclusions:**

The relationship between different types of physical activity and female infertility varies. We acknowledge the potential influence of confounding variables on this relationship. However, we have already adjusted for these potential variables in our analysis. Therefore, our findings suggest that appropriate recreational activity programs are essential for promoting reproductive health in women of reproductive age. Nevertheless, it is important to note that the observed association does not imply causality. Given the limitations of cross-sectional studies, further prospective cohort studies are needed to explore the causal relationship while accounting for additional confounding factors.

**Supplementary Information:**

The online version contains supplementary material available at 10.1186/s12958-024-01234-6.

## Introduction

Infertility is defined as the inability to achieve a clinical pregnancy following 12 months of regular, unprotected sexual intercourse [1]. It is estimated to impact millions of individuals and couples across the globe. The worldwide prevalence of infertility varies between 9% and 18%, showing a rising trend in recent years [2, 3]. In the United States, approximately 15% of couples experience infertility [4]. Infertility not only impacts patients’ ability to fulfill their reproductive needs but also poses an increased risk of developing reproductive cancers and metabolism-related diseases [5, 6]. Furthermore, it can result in profound psychological and social distress, along with significant financial burdens for the patients [7, 8].

Physical activity (PA) is characterized as any bodily movement generated by skeletal muscles that necessitates the expenditure of energy [9]. Physical activity encompasses three primary categories: work activities, recreational activities, and transport-related activities, playing a crucial role in various aspects of daily life. As a daily lifestyle, physical activity has been found to have an important impact on female reproductive function [10–12]. The American College of Obstetricians and Gynecologists (ACOG) recommends that women who are planning to conceive engage in a minimum of 150 min of moderate physical activity per week [13]. However, the guideline lacks detailed information regarding how variations in the type, intensity, or duration of physical activity might influence fertility status. To date, numerous studies have investigated the relationship between physical activity and infertility, yet their findings remain inconclusive and controversial.

A study conducted in 2009 suggested a positive association between elevated levels of physical activity and increased risk of infertility [14]. Conversely, another study proposed that insufficient physical activity could also have detrimental effects on fertility [15]. However, the majority of studies have reported no significant correlation between physical activity and infertility [16–19]. These conflicting findings not only generate controversy but also underscore the need for independent estimate of the relationship between different types of physical activity and infertility. Therefore, we conduct a cross-sectional study using the National Health and Nutrition Examination Survey (NHANES) database from 2013 to 2020 to explore the potential association between different forms of physical activity (PA) and the risk of infertility in reproductive age women.

## Methods

### Data source and study population

**Fig. 1:**
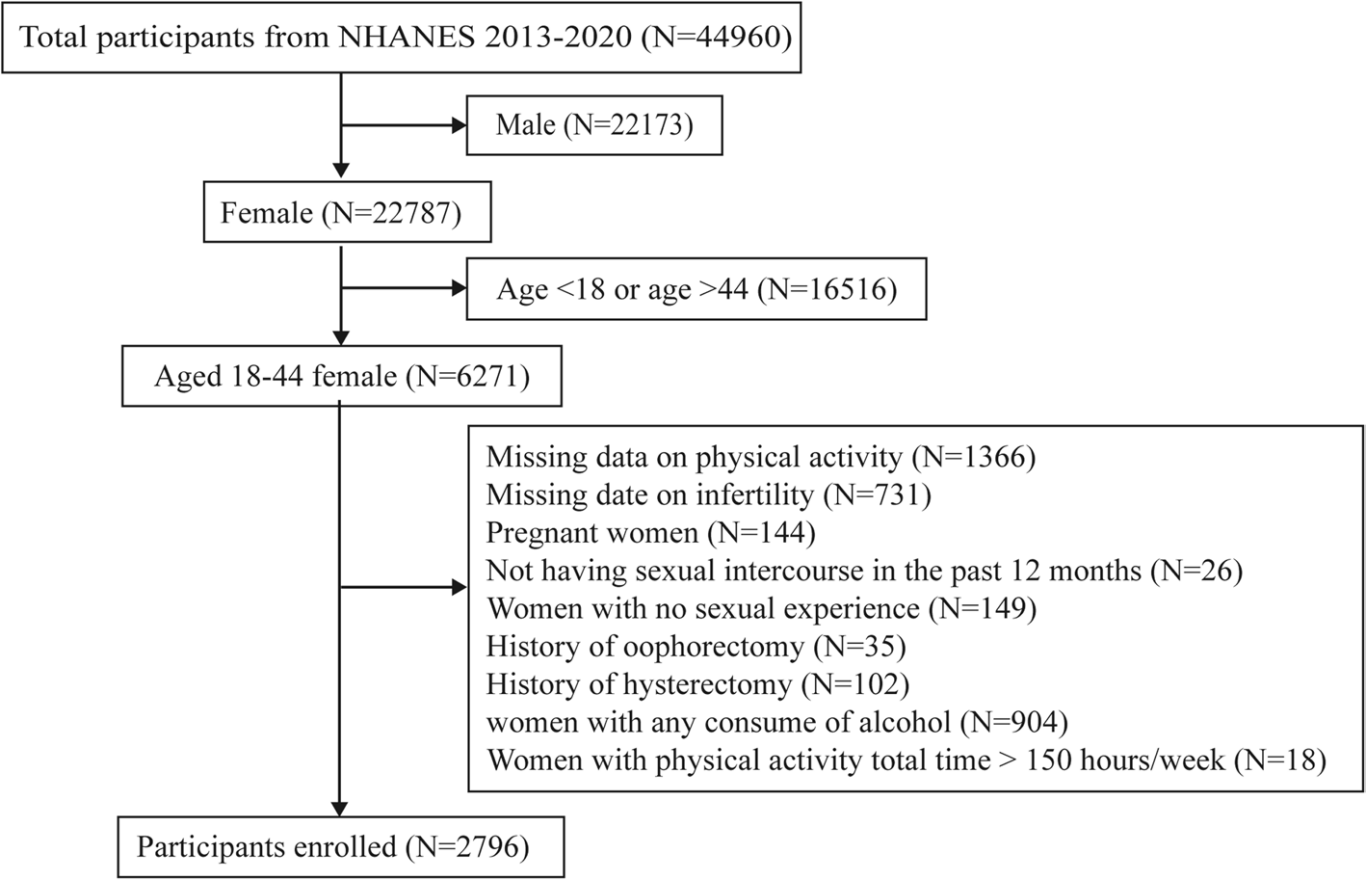
Flow chart for participants recruitment, NHANES 2013–2020

We obtained data from the NHANES, which is conducted by the National Center for Health Statistics (NCHS), a part of the Centers for Disease Control and Prevention (CDC). The data included information from four survey cycles spanning the years 2013–2014, 2015–2016, 2017–2018, and 2019–2020, with a total of 44,960 participants from the United States. We selected a final sample of 2,796 participants based on the following exclusion criteria: (1) Male; (2) Age < 18 or age > 44; (3) Missing data on physical activity; (4) Missing data on infertility; (5) Pregnant women; (6) Not having sexual intercourse in the past 12 months; (7) Women with no sexual experience; (8) Women with a history of oophorectomy or hysterectomy; (9) Women with any consume of alcohol; (10) Women with abnormal extreme values (> 150 h/week) for physical activity total time. The participant recruitment flow chart is shown in Fig. 1. All study methods in NHANES were conducted in accordance with the Declaration of Helsinki and the NHANES database is publicly accessible and allows other researchers to replicate the study, so no additional ethical approval is required. The study design and data from the NHANES can be accessed at https://www.cdc.gov/nhcs/nhanes/.

### Main variables

Data on PA from the NHANES database consists of three components self-reported from the Physical Activity Questionnaire: work activity, recreational activity, and walk or bicycle for transportation. Work activity was defined as paid or unpaid work, housework, and yard work. Recreational activity is related to sports, fitness and recreation. Walk or bicycle for transportation means walking or bicycling for travel, such as on the way to school, shopping, or work. The types of PA were further subdivided into moderate and vigorous activity, where vigorous activity was directed to induce substantial increases in heart rate and respiration. Participants were queried regarding the duration of time allocated to each category of PA during a typical week. Detailed information for collecting data on physical activity can be accessed through the NHANES website: https://wwwn.cdc.gov/nchs/nhanes/Default.aspx. According to the calculation of energy expenditure rate in the Compendium of Physical Activities, [20] we converted vigorous PA time to moderate PA time in a ratio of 2:1. In the subsequent study, the time spent in various types of PA was considered both as a continuous and categorical variable, with the categories grouped into tertiles based on the distribution.

Data on infertility were obtained from the NHANES Reproductive Health Questionnaire (RHQ074). The question was, “Have you ever attempted to become pregnant over a period of at least a year without becoming pregnant?“. Participants who answered “yes” would be considered infertile.

### Covariates

Covariates were collected including age (RIDAGEYR), race (RIDRETH3), body mass index (BMXBMI), educational level (DMQ.141), poverty-to-income ratio (INDFMPIR), smoking status (SMQ.040), marital status (DMDMARTZ), age at menarche (RHQ010), menstrual regularity (RHQ031), history of birth control pills using (RHQ420), history of hormones using (RHQ540), history of hypertension (BPQ020) and diabetes (DIP010). Race, education level, marital status, menstrual regularity, history of birth control pills using, history of hormones using, history of hypertension and diabetes were considered as categorical variables, and age, body mass index (BMI), poverty-to-income ratio (PIR), age at menarche were treated as continuous variables. Individuals with a history of smoking were classified as never smokers, former smokers or current smokers. Information of alcohol consumption (g/day) was also collected and participants with any consume of alcohol (daily alcohol consumption > 0 g/d) were excluded from this study.

### Statistical analysis

Appropriate weights were were employed during data analysis to ensure the conclusions reflect the broader U.S. population accurately. Participants were stratified into two groups based on infertility status, and their baseline clinical characteristics were delineated. For continuous variables with normal distribution, data are presented in the form of “Mean ± SD” with *p*-values obtained by t-test. For continuous variables with abnormal distribution, data are presented in the form of “Median (Q1-Q3)” with *p*-values obtained by Mann-Whitney U test. For categorical variables, data are presented as in the form of “sample size (%)” with *p*-value obtained by χ2 test. The logistic regression model was constructed to analyze the association between PA and infertility. Firstly, PA was analyzed as a continuous variable, and then PA was divided into three groups according to tertiles to further verify the association between PA and the probability of infertility. We presented different adjusted models to assess the association between PA and infertility according to the recommendations of Strengthening the Reporting of Observational Studies in Epidemiology (STROBE) statement [21]. Covariates need to be adjusted when they met the following criteria: (1) Covariate when was included or excluded from the model, the odd ratio changes by at least 10%; [22] (2) Covariate was associated with both PA and the probability infertility based on clinical practice; and (3) Covariate was adjusted in previous similar studies [23, 24]. The nonlinear relationship between recreational activity and female infertility was explored by smooth curve fittings. In order to determine whether the threshold existed or not, we performed a loglikelihood ratio test on the one-line (non-segmented) model according to the piecewise regression model. In addition, the subgroup analyses were performed using stratified linear regression models. Tests for effect modification by subgroup used interaction terms between subgroup indicators, followed by the likelihood ration test. Data analysis was performed using R (The R Foundation; http://www.r-project.org; version 4.2.0) and EmpowerStats (www.empowerstats.net, X&Y solutions, Inc. Boston, Massachusetts). A two-sided *P* value of less than 0.05 was considered to indicate statistical significance.

## Results

### The selection of participates

As shown in Fig. 1, the total number of participants in the NHANES program from 2013 to 2020 was 44,960. Participants who were male (*n* = 22,173), with age < 18 or age > 44 (*n* = 16,516), with missing data on physical activity (*n* = 1366) or infertility (*n* = 731), pregnant (*n* = 144), not having sexual intercourse in the past 12 months (*n* = 26), with no sexual experience (*n* = 149), had a history of oophorectomy (*n* = 33) or hysterectomy (*n* = 102), current drinker (*n* = 904) and with PA total time > 150 h/week were excluded, leaving 2796 participants for subsequent analysis.

### Baseline characteristics of participants

The baseline characteristics of the study population are shown in Table 1. There were 2483 participants in the fertile group and 313 in the infertile group. Compared with the fertile group, the infertile group had an older age (33.91 years vs. 29.99 years, *P* < 0.0001), a higher BMI (32.15 kg/m^2^ vs. 28.88 kg/m^2^, *P* = 0.0063), a higher proportion of individuals with previous hormone use (9.87% vs. 2.07%, *P* = 0.0075), as well as a higher prevalence of diabetes (19.76% vs. 11.49%, *P* = 0.0362) and hypertension (9.11% vs. 2.80%, *P* < 0.0001).

**Table 1:** Weighted demographic characteristics of selected participants from the NHANES 2013–2020

	Fertile	Infertile	*P*-value
Numbers of participants	2483	313	
Recreational activity time (hours/week)	7.26 (6.43, 8.09)	8.77 (6.68, 10.86)	0.21
Work activity time (hours/week)	20.27 (18.78, 21.76)	27.64 (20.00, 35.27)	0.05
Walk or bicycle time (hours/week)	4.22 (3.45, 4.98)	3.31 (2.45, 4.16)	0.10
Age (years)	29.99 (29.37, 30.61)	33.91 (32.34, 35.47)	< 0.01
Age (%)			< 0.01
< 30 years	50.05 (45.62, 54.48)	29.20 (20.94, 39.12)	
30–35 years	18.15 (15.22, 21.49)	19.18 (12.05, 29.15)	
≥ 35 years	31.80 (27.80, 36.08)	51.61 (40.00, 63.05)	
Race (%)			0.90
Non-Hispanic Black	14.42 (11.23, 18.32)	14.47 (9.39, 21.63)	
Non-Hispanic White	53.24 (47.00, 59.37)	56.04 (43.87, 67.52)	
Mexican American	14.59 (11.14, 18.88)	12.16 (6.62, 21.28)	
Others	17.75 (14.76, 21.20)	17.34 (11.06, 26.12)	
BMI (kg/m^2^)	28.88 (28.18, 29.58)	32.15 (29.99, 34.30)	< 0.01
BMI (%)			< 0.01
< 25 kg/m^2^	36.81 (32.78, 41.04)	28.37 (18.67, 40.60)	
25–30 kg/m^2^	26.72 (23.80, 29.87)	14.89 (7.80, 26.58)	
≥ 30 kg/m^2^	36.47 (32.96, 40.13)	56.74 (43.64, 68.96)	
Educational level (%)			0.74
Less than 9th grade	2.07 (1.29, 3.29)	1.41 (0.40, 4.83)	
High school or equivalent	27.42 (23.09, 32.23)	25.55 (18.11, 34.76)	
College or over	70.51 (65.60, 74.98)	73.04 (63.65, 80.74)	
PIR	2.56 (2.36, 2.75)	2.67 (2.38, 2.97)	0.53
Smoking status (%)			0.05
Never	71.99 (69.07, 74.74)	63.68 (57.52, 69.43)	
Former	10.22 (8.49, 12.25)	14.88 (9.66, 22.22)	
Current	17.79 (15.45, 20.39)	21.44 (15.59, 28.74)	
Marital status (%)			0.15
Widowed/Divorced/Separated/Never Married	40.63 (36.71, 44.67)	31.57 (21.04, 44.42)	
Married/Living with Partner	59.37 (55.33, 63.29)	68.43 (55.58, 78.96)	
Age at menarche (years)	12.52 (12.38, 12.67)	12.43 (12.06, 12.79)	0.63
Menstrual regularity (%)			0.98
No	7.03 (5.20, 9.43)	7.07 (3.58, 13.51)	
Yes	92.97 (90.57, 94.80)	92.93 (86.49, 96.42)	
History of birth control pills using (%)			0.63
No	29.76 (26.21, 33.57)	27.39 (19.17, 37.49)	
Yes	70.24 (66.43, 73.79)	72.61 (62.51, 80.83)	
History of hormones using (%)			< 0.01
No	97.93 (95.81, 98.99)	90.13 (74.63, 96.59)	
Yes	2.07 (1.01, 4.19)	9.87 (3.41, 25.37)	
Hypertension (%)			0.04
No	88.51 (85.72, 90.82)	80.24 (70.17, 87.51)	
Yes	11.49 (9.18, 14.28)	19.76 (12.49, 29.83)	
Diabetes (%)			< 0.01
No	97.20 (96.44, 97.80)	90.89 (86.19, 94.10)	
Yes	2.80 (2.20, 3.56)	9.11 (5.90, 13.81)	

Data in the table: For continuous variables: survey-weighted mean (95% confidence interval), *P*-value was by survey-weighted linear regression (svyglm). For categorical variables: survey-weighted percentage (95% confidence interval), *P*-value was by survey-weighted Chi-square test (svytable)

*BMI* Body mass index, *PIR* Poverty-to-income ratio

### The association between time of various PA and infertility

Univariable and multivariable logistic regression models were applied to explore the association between different types of PA duration and female infertility (Table 2). In the fully adjusted model (adjusted for age, race, BMI, educational levels, marital status, smoking status, history of hormones using, hypertension and diabetes), recreational activity and work activity were significantly associated with infertility (OR = 1.04, 95% CI: 1.01 to 1.08, *P* = 0.01; OR = 1.01, 95% CI: 1.00 to 1.02, *P* = 0.02). When treating recreational activity time as categorical variables, a similar trend was seen (p for the trend was 0.02), but work activity became not significantly associated with infertility. In addition, walking or bicycle was not associated with infertility in any of the three models. We also performed sensitivity analyses by dividing various PA time into quartiles, and the results remained stable (Supplementary Table 1).

**Table 2:** Relationship between physical activity (tripartite grouping) and female infertility in different models

Exposure	Crude Model		Model I		Model II		Model III	
	OR (95% CI)	*P* value	OR (95% CI)	*P* value	OR (95% CI)	*P* value	OR (95% CI)	*P* value
Recreational activity time (hours/week)								
(continuous)	1.02 (0.99, 1.05)	0.20	1.03 (1.00, 1.02)	0.04	1.04 (1.01, 1.08)	0.01	1.05 (1.01, 1.08)	0.02
(tertile)								
≤ 3.00	Ref.		Ref.		Ref.		Ref.	
3.00–7.50	0.83 (0.42, 1.65)	0.60	0.87 (0.43, 1.76)	0.71	0.93 (0.46, 1.86)	0.84	0.90 (0.47, 1.73)	0.76
≥ 7.50	1.73 (0.93, 3.20)	0.09	1.98 (1.02, 3.85)	0.05	2.37 (1.22, 4.59)	0.02	2.38 (1.02, 4.71)	0.02
P for trend		0.10		0.06		0.02		0.03
Work activity time (hours/week)								
(continuous)	1.01 (1.00, 1.02)	0.02	1.01 (1.00, 1.02)	0.01	1.01 (1.00, 1.02)	0.01	1.01 (1.00, 1.02)	0.02
(tertile)								
≤ 6.00	Ref.		Ref.		Ref.		Ref.	
6.00–24.00	1.26 (0.65, 2.44)	0.49	1.27 (0.66, 2.45)	0.48	1.30 (0.66, 2.56)	0.45	1.18 (0.55, 2.50)	0.67
≥ 24.00	1.68 (0.83, 3.42)	0.16	1.63 (0.78, 3.37)	0.20	1.65 (0.79, 3.44)	0.19	1.58 (0.72, 3.45)	0.26
P for trend		0.16		0.20		0.20		0.26
Walk or bicycle time (hours/week)								
(continuous)	0.97 (0.92, 1.01)	0.18	0.98 (0.94, 1.03)	0.46	0.98 (0.93, 1.03)	0.45	0.98 (0.94, 1.03)	0.51
(tertile)								
≤ 1.50	Ref.		Ref.		Ref.		Ref.	
1.50–3.50	1.62 (0.53, 4.91)	0.40	1.49 (0.52, 4.28)	0.46	2.14 (0.75, 6.10)	0.17	2.08 (0.72, 6.00)	0.19
≥ 3.50	0.65 (0.24, 1.77)	0.41	0.73 (0.28, 1.92)	0.53	0.77 (0.29, 2.05)	0.60	0.80 (0.29, 2.20)	0.67
P for trend		0.38		0.54		0.60		0.70

Model I adjusted for age and race

Model II adjusted for age, race, BMI, educational level, marital status and smoking status

Model III further adjusted for history of hormones using, hypertension, diabetes

*OR* Odds radio, *CI* Confidence interval, *Ref.* Reference, *BMI* Body mass index

### The analyses of non-linear relationship between recreational activity time and infertility

**Fig. 2:**
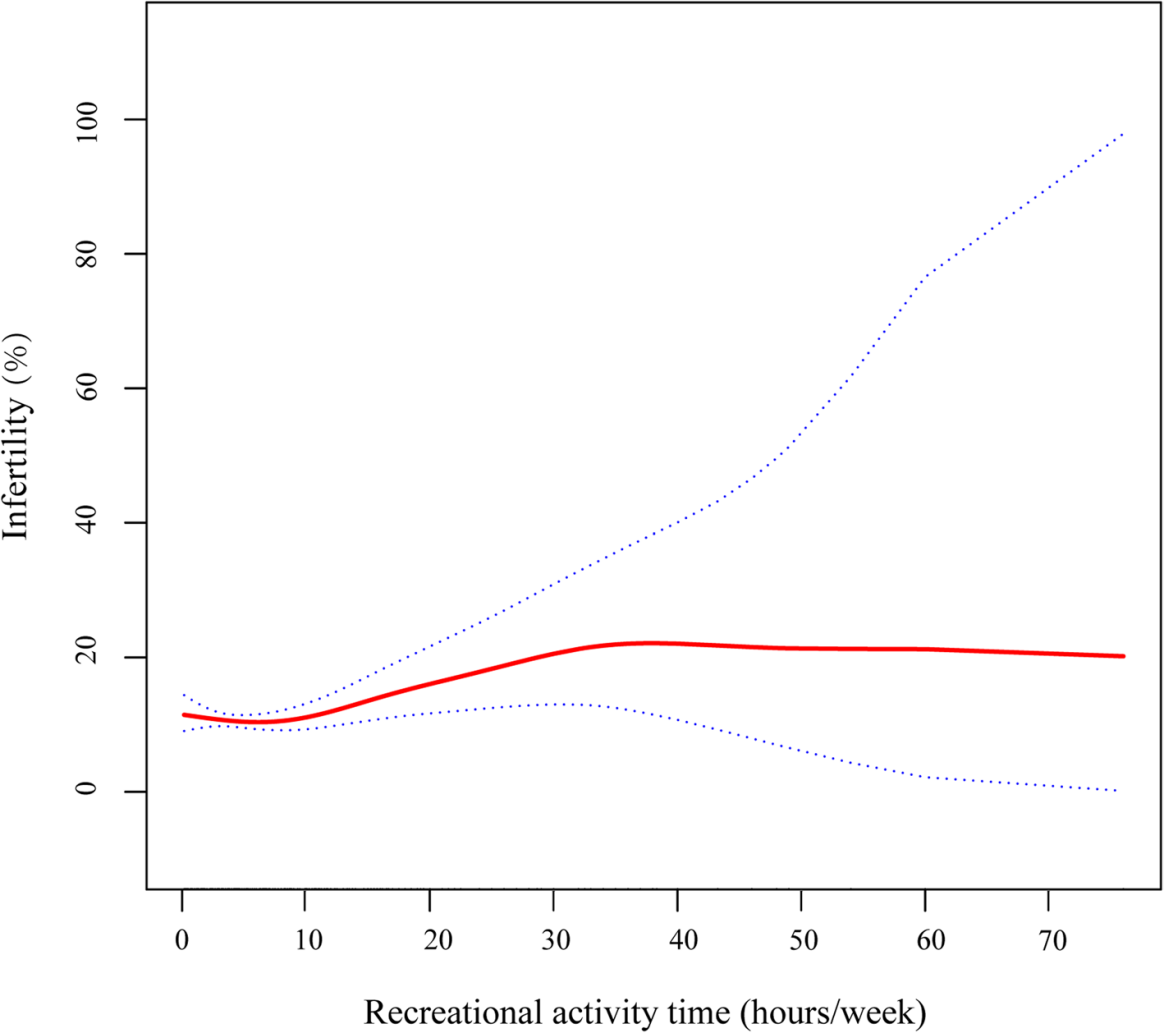
Adjusted associations of recreational activity time with female infertility. A non-linear relationship was found. Red line represents the smooth curve fit between variables. Blue bands represent the 95% of confidence interval from the fit. Adjusted: age, race, BMI, educational level, marital status and smoking status. BMI, body mass index

We additionally explored the potential for a non-linear relationship between recreational activity duration and infertility through the utilization of smooth curve fits (Fig. 2). After adjusting age, race, BMI, educational level, marital status and smoking status, we found that the relationship between recreational activity time and female infertility was nonlinear. Using a two-piecewise linear regression model, we were able to identify that the inflection point was located at 5.38 h/week (Table 3). On the left side of the inflection point, there was no significant association between recreational activity time and infertility (OR = 0.93, 95% CI: 0.86 to 1.02, *P* = 0.1146), but on the right side of the inflection point, there was a positive association between recreational activity time and the risk of infertility (OR = 1.04, 95% CI: 1.02 to 1.06, *P* = 0.0008).

**Table 3:** Threshold effect analysis of physical activity and female infertility using two-piecewise linear regression

Models	Effect size (OR)	95% CI	*P* value
Recreational activity time (hours/week)			
Model 1			
One line effect	1.02	1.00 to 1.04	0.02
Model 2			
Inflection point			
< 5.83	0.93	0.86 to 1.02	0.11
≥ 5.83	1.04	1.02 to 1.06	< 0.01
*P* value for LRT test*			0.03

Model 1, linear analysis; Model 2, non-linear analysis

Adjusted: age, race, BMI, educational levels, marital status and smoking status

*OR* Odds radio, *CI* Confidence interval, *BMI* Body mass index, *LRT* Logarithm likelihood radio test

*P < 0.05 indicates Model 2 is significantly different from Model 1

### The results of subgroup analyses

**Fig. 3:**
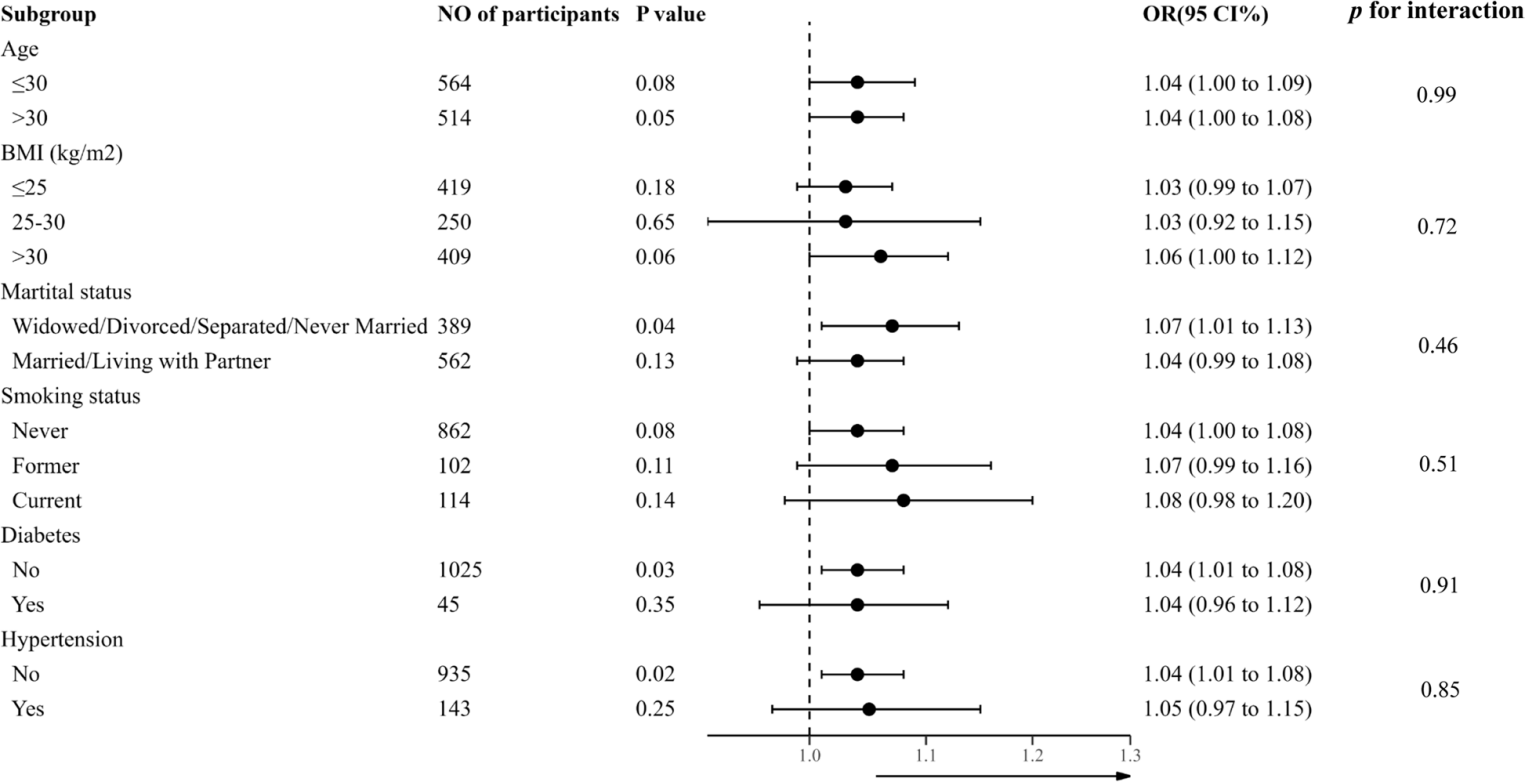
Effect size of recreational activity time on female infertility in subgroups analysis. Each stratification adjusted for all the factors (age, race, BMI, educational level, marital status and smoking status) except the stratification factor itself. OR, odds radio; CI, confidence interval; BMI, body mass index

To further test the stability of the results, we performed subgroup analyses by age, BMI, marital status, smoking status, history of diabetes and hypertension as shown in Fig. 3. After adjusting age, race, BMI, educational level, marital status and smoking status, the test for interactions were not significant in each subgroup (all *P* values for interactions were larger than 0.05).

## Discussion

Our research identified a non-linear association between recreational activity time and the risk of female infertility, pinpointing an inflection point at 5.83 h/week (moderate intensity). Beyond this inflection point, as the duration of recreational activity extends, the risk of infertility correspondingly escalates (OR = 1.04, 95% CI: 1.02 to 1.06, *P* < 0.01). However, there was no similar association between work activity time, walking or bicycle time and infertility. In the results of the subgroup analysis, we observed that the association between recreational activity duration and infertility remained unaffected by these stratified variables, demonstrating its stability. In the study population that met our exclusion criteria, after adjusting for age, race, BMI, educational levels, marital status and smoking status, our finding suggested different associations between different types of physical activity and infertility.

Distinguishing our study from previous research, we specifically excluded female participants who were current alcohol consumers. This decision was based on the clear understanding that habitual alcohol consumption negatively impacts female reproductive function [25] and is typically avoided by women intending to conceive. Based on this exclusion criterion, our study identified a positive correlation between prolonged periods of recreational activity and the risk of infertility. This is consistent with the findings of a previous study, which demonstrated that high intensity and frequency of physical activity have a negative impact on female reproductive health [14]. However, other studies have discovered no significant link between physical activity and female infertility, [16–19] or have indicated that physical activity may actually act as a protective factor against infertility [15]. We believe that the divergence in research findings is likely due to the studies not considering the independent effects that different types of physical activity may have on the human body, as well as the lack of adjustment for certain confounding factors or the selection of appropriate inclusion criteria.

To the best of our knowledge, our study represents the first attempt to explore the relationship between various forms of physical activity and infertility. Our findings indicate that the relationship between various types of physical activity and infertility is not uniform. In our study, recreational activities had a more stable association with infertility than work activities, whereas traffic-related activities had no significant association with infertility. Two prior studies have similarly indicated that various types of physical activity exert distinct effects on the body, which supported the physical activity paradox [26, 27]. The variation observed might be attributed to self-determined motivation [28]. Recreational activity represent those chosen by individuals to engage in during their leisure time, whereas work activity are obligations that individuals must fulfill during their working hours. Consequently, recreational activity possess a more subjective nature compared to work activity. Moreover, distinct types of physical activity exhibit varying characteristics. Recreational activity predominantly involve high-intensity and short-duration exercises, whereas work activity tend to consist of prolonged periods of low-intensity and static tasks.

Engaging in high-intensity recreational activities for prolonged durations may result in infertility through various mechanisms. On the one hand, high-intensity physical activity may interact with additional psychosocial and metabolic stressors, prompting physiological stress responses. This can disrupt the pulsatile secretion of hypothalamic gonadotropin-releasing hormone (GnRH), which, via the hypothalamic-pituitary-ovarian (HPO) axis, impedes the production of estrogen and progesterone - pivotal hormones for ovulation and conception [29]. On the other hand, it can induce infertility by causing negative energy balance and impeding the necessary processes for ovulation [30].

Our study possesses several notable strengths. Firstly, we leveraged data from the NHANES database, which offers comprehensive coverage across all regions of the United States and ensures strong representativeness. Secondly, our investigation separately examined the relationship between various types of physical activity and infertility, uncovering a non-linear correlation between recreational activity time and infertility. Thirdly, by employing threshold effect analysis, we identified the inflection point of moderate intensity recreational activity time at 5.83 h/week, thereby offering valuable recommendations for the weekly exercise duration for women of childbearing age. Lastly, through subgroup analysis, we revealed that the relationship between recreational activity duration and infertility remained stable and unaffected by the stratified variables.

However, there are some limitations to our study. First, despite revealing a correlation between physical activity and infertility, establishing causation is not possible due to the cross-sectional nature of the study. Future prospective studies are required to investigate the causal relationship between the two factors. Second, our study is based on self-reported data, which includes information on infertility and physical activity. It is important to consider that self-reporting may introduce recall bias, as women might either overestimate or underestimate their exercise levels and misjudge their infertility status. Third, the NHANES dataset did not contain information on the precise length of infertility or the fertility status of their partners. Fourth, due to the lack of data on conditions such as polycystic ovary syndrome and endometriosis, which can have an impact on female fertility, within the NHANES database, we cannot exclude the influence of these potential factors on our results. Lastly, as the dataset originates from a nationwide survey in the United States, further validation is needed to confirm its generalizability across different racial groups.

In conclusion, our findings indicate a non-linear correlation between recreational activity duration and infertility, and the relationship between different types of physical activity and female infertility varies, which offering valuable insights for establishing healthy physical activity guidelines for women of childbearing age. However, because this study was a cross-sectional study, more prospective cohort studies are needed in the future to explore causality.

### Supplementary Information

Supplementary Material 1.

## Data Availability

The datasets presented in this study can be found in online repositories. The names of the repository/repositories and accession number(s) can be found below: https://www.cdc.gov/nchs/nhanes/index.htm.
