# Supplementary material for: Effects of physical activity on infertility in reproductive females
Source: Reprod Biol Endocrinol. 2024 May 29;22:62. doi: 10.1186/s12958-024-01234-6 (PMC11134942; doi:10.1186/s12958-024-01234-6)
Supplement: Supplementary file 1 — Supplementary Material 1. [file 12958_2024_1234_MOESM1_ESM.docx]

**Supplementary Table 1 Relationship between physical activity (tripartite grouping) and female infertility in different models.**

| Exposure | Crude Model | |  | Model Ⅰ | |  | Model Ⅱ | |  | Model Ⅲ | |
| --- | --- | --- | --- | --- | --- | --- | --- | --- | --- | --- | --- |
|  | OR (95% CI) | P value |  | OR (95% CI) | P value |  | OR (95% CI) | P value |  | OR (95% CI) | P value |
| Recreational activity  time  (hours/week) |  |  |  |  |  |  |  |  |  |  |  |
| (continuous) | 1.02 (0.99, 1.05) | 0.20 |  | 1.03 (1.00, 1.06) | 0.04 |  | 1.01 (1.00, 1.02) | 0.01 |  | 1.05 (1.01, 1.08) | 0.02 |
| (quartile) |  |  |  |  |  |  |  |  |  |  |  |
| ≤ 2.00 | Ref. |  |  | Ref. |  |  | Ref. |  |  | Ref. |  |
| 2.00 - 5.00 | 0.95 (0.53, 1.71) | 0.86 |  | 0.90 (0.47, 1.75) | 0.77 |  | 0.96 (0.52, 1.76) | 0.89 |  | 0.94 (0.51, 1.73) | 0.83 |
| 5.00 - 9.50 | 0.66 (0.28, 1.52) | 0.33 |  | 0.68 (0.29, 1.59) | 0.38 |  | 0.70 (0.30, 1.65) | 0.42 |  | 0.69 (0.29, 1.67) | 0.42 |
| ≥ 9.50 | 1.78 (0.89, 3.57) | 0.11 |  | 2.14 (1.01, 4.50) | 0.05 |  | 2.55 (1.24, 5.27) | 0.02 |  | 2.57 (1.19, 5.53) | 0.02 |
| P for trend |  | 0.18 |  |  | 0.09 |  |  | 0.04 |  |  | 0.05 |
| Work activity  time  (hours/week) |  |  |  |  |  |  |  |  |  |  |  |
| (continuous) | 1.01 (1.00, 1.02) | 0.02 |  | 1.01 (1.00, 1.02) | 0.01 |  | 1.01 (1.00, 1.02) | 0.01 |  | 1.01 (1.00, 1.02) | 0.02 |
| (quartile) |  |  |  |  |  |  |  |  |  |  |  |
| ≤ 4.00 | Ref. |  |  | Ref. |  |  | Ref. |  |  | Ref. |  |
| 4.00 - 15.00 | 0.75 (0.35, 1.61) | 0.46 |  | 0.70 (0.32, 1.56) | 0.39 |  | 0.72 (0.33, 1.58) | 0.41 |  | 0.53 (0.23, 1.26) | 0.16 |
| 15.00 - 35.00 | 0.86 (0.39, 1.92) | 0.72 |  | 0.80 (0.36, 1.79) | 0.59 |  | 0.82 (0.38, 1.78) | 0.62 |  | 0.67 (0.31, 3.45) | 0.32 |
| ≥ 35.00 | 1.69 (0.76, 3.75) | 0.20 |  | 1.59 (0.70, 3.64) | 0.28 |  | 1.64 (0.72, 3.76) | 0.25 |  | 1.43 (0.61, 3.40) | 0.42 |
| P for trend |  | 0.16 |  |  | 0.22 |  |  | 0.19 |  |  | 0.28 |
| Walk or  Bicycle time (hours/week) |  |  |  |  |  |  |  |  |  |  |  |
| (continuous) | 0.97 (0.92, 1.01) | 0.18 |  | 0.98 (0.94, 1.03) | 0.46 |  | 0.98 (0.93, 1.03) | 0.45 |  | 0.98 (0.94, 1.03) | 0.51 |
| (quartile) |  |  |  |  |  |  |  |  |  |  |  |
| ≤ 1.00 | Ref. |  |  | Ref. |  |  | Ref. |  |  | Ref. |  |
| 1.00 - 2.25 | 1.54 (0.43, 5.55) | 0.51 |  | 1.53 (0.42, 5.58) | 0.52 |  | 2.15 (0.55, 8.36) | 0.28 |  | 2.14 (0.52, 10.70) | 0.46 |
| 2.25 - 5.00 | 2.18 (0.54, 8.82) | 0.28 |  | 2.21 (0.56, 8.67) | 0.26 |  | 3.17 (0.77, 13.03) | 0.12 |  | 3.27 (0.79, 13.47) | 0.53 |
| ≥ 5.00 | 1.01 (0.76, 1.40) | 0.99 |  | 1.11 (0.33, 3.77) | 0.87 |  | 1.36 (0.38, 4.89) | 0.64 |  | 1.53 (0.38. 6.11) | 0.55 |
| P for trend |  | 0.83 |  |  | 0.67 |  |  | 0.48 |  |  | 0.42 |

Model Ⅰ adjusted for age and race.

Model Ⅱ adjusted for age, race, BMI, educational level, marital status and smoking status.

Model Ⅲ further adjusted for history of hormones using, hypertension, diabetes.

OR, odds radio; CI, confidence interval; Ref., reference; BMI, body mass index.
